# Artificial Anæstheisa

**Published:** 1890-02

**Authors:** 


ARTIFICIAL ANÆSTHESIA.—A Manual of Anæsthetic Agents and their employment in the Treatment of Disease. By Laurence Turnbull, M. D., PH. G., Aural Surgeon to the Jefferson Medical College Hospital, Etc., Etc. Third edition, revised and enlarged, with illustrations. Philadelphia: P. Blackiston, Son & Co., 1890.

The author has in this new edition, made a thorough revision of the whole subject of Artificial Anæsthesia, and presented a careful and conscientious study of each anæsthetic agent, defining its natural and physiological characteristics and peculiarities.

He has also shown that much of the risk to life is prevented by the discovery of true and positive local anæsthetics, and by their careful use in all minor operations in surgery. He also asserts that all hopes have passed away—for the time being at least, that any one of the systemic anæsthetics is absolutely free from risk to life, as anæsthesia carried to the effect of a profound impression on the human subject, sufficient for a capital operation, is but a step from death. The author has succeeded in making his work a scientific, yet practical and safe guide in the administration of anæsthetic agents, and added much to the matter presented in the former edition.

